# Home-based functional walking program for advanced cancer patients receiving palliative care: a case series

**DOI:** 10.1186/1472-684X-12-22

**Published:** 2013-05-11

**Authors:** Sonya S Lowe, Sharon M Watanabe, Vickie E Baracos, Kerry S Courneya

**Affiliations:** 1 Department of Symptom Control and Palliative Care, Cross Cancer Institute, 11560 University Avenue, Edmonton, Alberta, T6G 1Z2, Canada; 2 Division of Palliative Care Medicine, Department of Oncology, University of Alberta, Alberta, Canada; 3 Physical Activity and Cancer, Physical Education & Recreation, University of Alberta, E-488 Van Vliet Centre, Edmonton, Alberta, T6G 2H9, Canada

**Keywords:** Physical activity, Palliative care, Cancer, Quality of life, Walking

## Abstract

**Background:**

Although meta-analyses have demonstrated that physical activity can positively impact quality of life outcomes in early stage cancer patients, it is not yet known whether these benefits can be extended to patients with advanced cancer. In a previous pilot survey of patients with advanced cancer with a median survival of 104 days, participants felt willing and able to participate in a physical activity intervention, and reported a strong preference for walking and home-based programming. Here, we report on the initial development and feasibility of a home-based functional walking program in patients with advanced cancer receiving palliative care.

**Methods:**

Nine adult patients were recruited from outpatient palliative care clinics and palliative home care. A pilot intervention trial was conducted over a 6-week period. The McGill Quality of Life Questionnaire (MQOL), Late Life Function and Disability Instrument (LLFDI), Edmonton Symptom Assessment System (ESAS), Seniors Fitness Test, four-test balance scale, and grip strength, were performed pre- and post-intervention. Participants wore *activ*PAL™ accelerometers to monitor ambulatory activity levels.

**Results:**

Of the nine recruited participants, three participants dropped out prior to baseline testing due to hospital admission and feeling overwhelmed, and three participants dropped out during the intervention due to severe symptoms. Only three participants completed the intervention program, pre- and post-intervention assessments: two reported improvements in total MQOL scores, yet all three shared an overall trend towards worsening symptom and total fatigue scores post-intervention. Two participants passed away within 90 days of completing the intervention.

**Conclusions:**

This case series demonstrates the challenges of a physical activity intervention in patients with advanced cancer receiving palliative care. Further feasibility research is required in this patient population.

**Trial registration:**

This study is registered under ClinicalTrials.gov as NCT00438620.

## Background

Among the most common distressing symptoms facing patients with advanced cancer is loss of physical function [1]. Its underlying aetiology is multifactorial, with increasing fatigue, muscle wasting and generalized debility all contributing to this phenomenon [2]. Loss of physical function impedes the patient’s ability to perform activities of daily living, and increases dependence on caregivers leading to additional emotional and psychological burden [3]. The importance of keeping mobile is linked to maintaining independence and overall quality of life [QoL] in patients with advanced cancer.

Increasing attention has been given to physical activity as a QoL intervention in cancer patients [4]. Physical activity interventions can improve cancer-related fatigue and physical functioning in early stage cancer patients [5,6]; however, these benefits have not been confirmed for patients at later stages of cancer. There is preliminary evidence that select patients with advanced cancer express willingness to participate in a physical activity intervention, with positive benefit on some supportive care outcomes [7].

Oldervoll et al. conducted a randomized controlled trial to examine the effects of an eight-week group exercise program versus usual care on 231 patients with advanced cancer with median survival of one year [8]. Analyses revealed no significant differences between groups in the primary endpoint of physical fatigue, however there were improvements in the intervention group in physical performance as measured by the shuttle walk and handgrip strength tests. Oldervoll et al. earlier acknowledged that patients who declined participation had identified limitations of fatigue, lack of mobility, and the burden of physically getting to the hospital gym where the exercise intervention took place [9]. Oldervoll et al. concluded that these limitations “might indicate a need for specially tailored interventions…in the form of home-based exercises adjusted for the individual patient” [10].

No home-based physical activity program has been validated for patients with advanced cancer receiving palliative care. Porock et al. conducted a pilot study of nine home hospice cancer patients who were administered a home-based program based on the Duke Energizing Exercise Plan, with a range of different physical activities prescribed according to the patient’s individual condition and tolerability; despite the trend towards increased QoL scores, the authors concluded that the optimal type of physical activity program for this population is still unknown [11].

We previously completed a pilot survey of fifty patients with advanced cancer with a median survival of 104 days; 92% of participants reported that they would be interested in and able to participate in a physical activity program [12]. Moreover, 84% of participants indicated a preference for a home-based individual (i.e. not group) physical activity program. Walking and resistance training were the top two activities endorsed by these participants, with 56% preferring to participate in up to 3 physical activity sessions per week [13].

Incorporating patient preferences is critical in designing an effective intervention [14] and may enhance recruitment and adherence, and potential benefits. Based on the preferences identified in our pilot survey and using a similar recruitment strategy, this study examined the initial development and feasibility of a home-based functional walking program in patients with advanced cancer receiving palliative care.

## Methods

### Setting and participants

The study was conducted between July to December 2007 at the Department of Symptom Control and Palliative Care, Cross Cancer Institute (CCI) and the Regional Palliative Home Care program (RPHCP) in Edmonton, Canada. Participants were diagnosed with progressive, incurable, and locally recurrent or metastatic cancer, and were receiving palliative care. Eligibility criteria included: 1) 18 years of age or older; 2) able to understand and speak English; 3) cognitive ability to participate (defined as a normal Folstein’s Mini Mental State Examination Score for patient’s age and education level [15]); and 4) clinician-estimated life expectancy of 3 to 12 months.

Participants were ineligible if they presented with: 1) Any absolute contraindications to physical activity [16]; and 2) Palliative Performance Scale (PPS) level of 30% or less [17]. Eligible participants were required to read and sign a consent form, which detailed the right to withdraw, confidentiality, and the risks and benefits of participating in the study.

### Consent

Ethical approval for the study was received from the Health Research Ethics Board of the University of Alberta and the Research Ethics Committee of the Alberta Cancer Board. Written informed consent was obtained from the patients for publication of this case report and any accompanying images. A copy of the written consent is available for review.

### Study design and recruitment

The study was a quasi-experimental pilot study using pre-post test design to provide preliminary data on the feasibility and outcomes of a six-week physical activity program. Consecutive patients were approached by a member of the health care team at both the RPHCP and CCI settings, and if interested in participating, they consented to be contacted by the study coordinator.

### Physical activity intervention

The intervention was a modified home-based functional walking program involving an individually prescribed walking plan and combination of muscle strengthening and balance retraining exercises [18,19]. The aerobic component required participants to perform daily walking, with duration and intensity individually prescribed based on the results of baseline physical function testing. For the strength component, participants performed individualized muscle strengthening and balance retraining exercises, three times per week on non-consecutive days (see Additional file 1: Appendix 1). A professional exercise therapist supervised all strength sessions in the participant’s home.

The mode, intensity (resistance) and duration of each strength exercise were based on the results of the participant’s baseline physical function testing. Variations on each strength exercise were provided for increasing levels of difficulty and to allow for individual prescription (see Additional file 2: Appendix 2). Ankle/wrist cuff weights and/or resistance bands were used to provide resistance during muscle strengthening and balance retraining exercises. Changes in number of exercises, sets and repetitions were made with the aim to progress to the desired exercise prescription as soon as safely possible. Five minutes of warm up and cool down exercises were performed before and after each strength session (see Additional file 1: Appendix 1).

### Objective assessment of physical functioning

Physical functioning was measured using six basic physical function parameters associated with functional tasks and activities that are significant in the everyday living of older adults [20]. Balance was assessed via a four-test balance scale [18] (see Additional file 3: Appendix 3). Grip strength was assessed using a handheld dynamometer. In addition to these standardized tests, the participant’s height, weight, body mass index, blood pressure, heart rate and oxygen saturation were measured. The study coordinator performed all physical functioning measurements in the patient’s home.

### Objective assessment of physical activity

Physical activity was recorded using the *activ*PAL™ accelerometer, which monitors triaxial movement in the form of lying or sitting, quiet standing and stepping [21]. The 20 gram, 35 × 53 × 7 millimetre unit is secured to the participant’s anterior mid-thigh using an adherent hydrogel PALstickie™ and participants were asked to remove the units when bathing or showering, and replace once the underlying skin is dried. Participants were asked to wear the unit for one baseline week prior to initiation of the intervention, and for the 6-week duration of the program. The *activ*PAL™ accelerometer has been validated in a number of clinical populations [22], and most recently has been tested in a study of 84 patients with thoracic cancer [23].

### Survey instrument

The survey instrument was administered once at baseline, and again post-intervention. The McGill Quality of Life Questionnaire (MQOL) [24] was used to assess quality of life; the MQOL has been found to be comprehensive, widely tested and valid across end-of-life populations [25]. Physical activity behaviour was assessed by items drawn from the Physical Activity Scale for the Elderly (PASE) which requires participants to recall their most common physical activities, including frequency, intensity and duration, performed over the past week [26]. The PASE was developed for assessment of community-dwelling, older adults and has been widely used and validated in various clinical populations [27,28]. For the purposes of the study, physical activity was defined as any bodily movement produced by the skeletal muscles that result in a substantial increase in energy expenditure over resting levels [29].

Patient-reported physical functioning was assessed by the function component of the abbreviated version of the Late-Life Function and Disability Instrument (LLFDI) [30]; the LLFDI has been widely used and validated in elderly populations [31].

Patient-reported symptoms were assessed using the Edmonton Symptom Assessment System (ESAS) [32], the Brief Fatigue Inventory (BFI), and the Hope Differential-Short Instrument (HDS), each of which has been respectively tested and validated in advanced cancer populations [33-35].

### Program feasibility

Program feasibility was assessed by the following: 1) recruitment rate, or the number of participants accrued as a proportion of those eligible, 2) retention rate, or the number of participants completing the post-intervention assessments, 3) adherence rate, or the number of sessions attended as a proportion of the maximum prescribed, and 4) patient safety, or number and type of adverse events.

## Results

### Sample characteristics and recruitment

**Figure 1:**
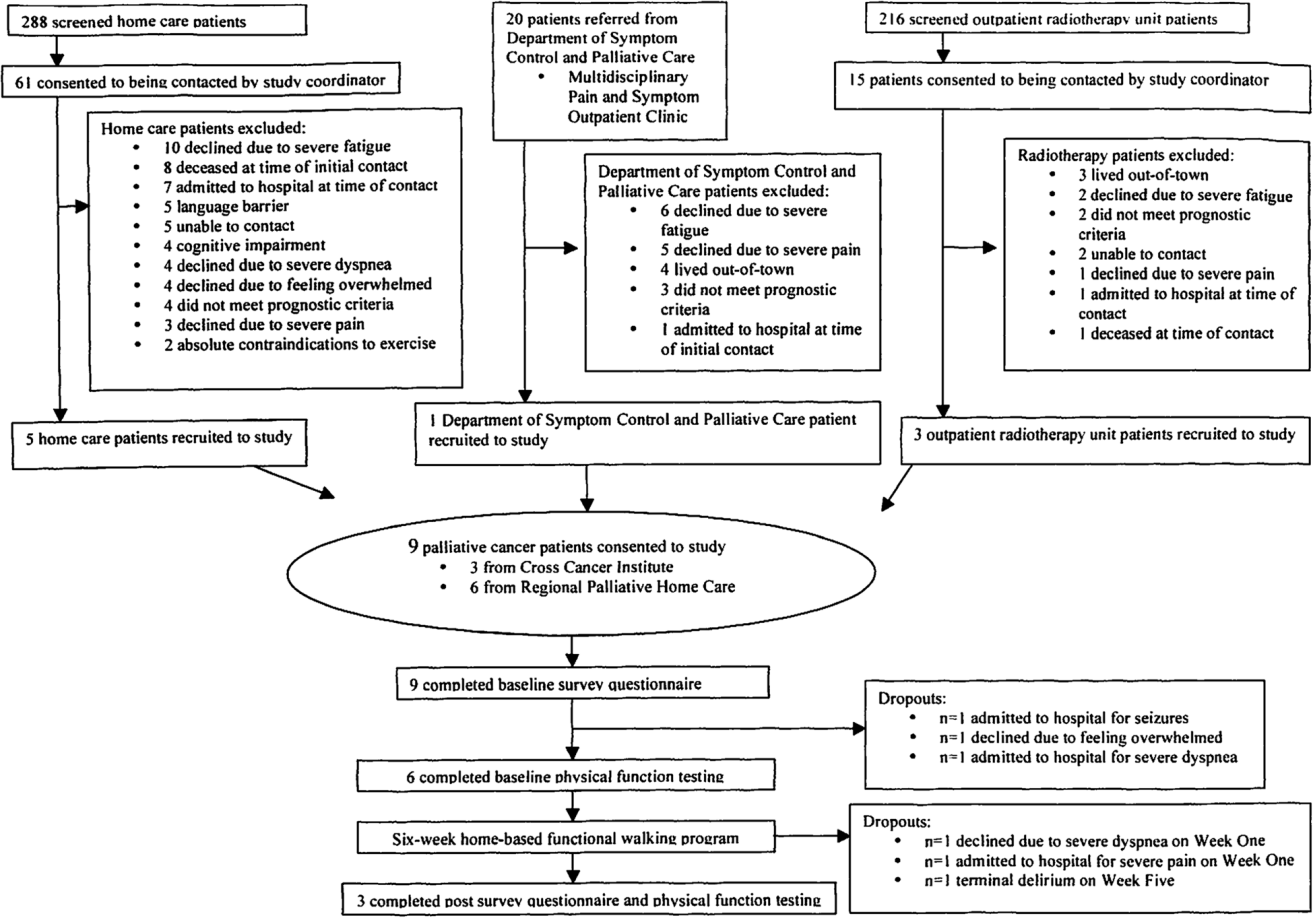
Flow of participants through the study.

Accrual was stopped early due to slower than expected accrual and higher than expected attrition. As shown by Figure 1, 16% (10/61) of home care patients who consented to being contacted by the study coordinator, declined due to severe fatigue; 8% (5/61) of RPHCP patients who consented to being contacted by the study coordinator, were recruited to the study. 30% (6/20) of Department of Symptom Control and Palliative Care patient referrals declined due to severe fatigue; 5% (1/20) of the remaining eligible patient referrals were recruited to the study. 20% (3/15) of outpatient radiotherapy unit patients who consented to being contacted by the study coordinator, did not meet inclusion criteria for the study because of out-of-town residence; 20% (3/15) of the remaining eligible patients were recruited to the study.

Of the 9 patients who consented to the study, 2 participants dropped out prior to baseline physical function testing because of admission to hospital, and 1 participant dropped out prior to baseline physical function testing because of feeling overwhelmed. Of the 6 patients who completed baseline physical function testing, 2 participants dropped out during Week One because of severe dyspnoea and pain, and 1 participant dropped out during Week Five because of terminal delirium. 3 participants who completed baseline physical function testing, also completed the intervention and post-intervention assessments.

Of the 9 patients who consented to the study, the mean age was 55 ± 5.7 years, 6/9 were female, and 5/9 were married or common law. Gastrointestinal cancer (n = 2), lung cancer (n = 2) and primary unknown (n = 2) were the most common diagnoses; the remaining diagnoses were head and neck cancer (n = 1), malignant melanoma (n = 1) and anaplastic oligodendroglioma (n = 1). 7/9 had metastatic disease, with liver (n = 4) being the most common site of metastases; 5/9 had two or more metastatic sites concurrently. At the time of obtaining consent, 6/9 patients had received palliative chemotherapy. Of the 9 patients who consented to the study, the median survival was 92 days from time of consent to time of death.

Given that only 3 participants completed the intervention and post-intervention assessments, inferential statistics were not possible and all accumulated data was reviewed descriptively. Hence the following 3 cases are presented to review the participants who completed the program.

#### Case 1

A 56 year-old man was diagnosed with cancer of unknown primary, with metastases to the lung, liver, bone and brain. He received a full course of palliative whole-brain radiotherapy, and daily dexamethasone was initiated. The patient was recruited to the study post-radiotherapy, and a summary of the participant’s baseline assessment is provided in Table 1. His most common reported physical activity over the past week was climbing stairs within his home, in order to access his bedroom and bathroom on the top floor.

**Table 1:** Outcome measures for case participants

**Outcome measure**		**Assessment**	**Case #1 Pre**	**Case #1 Post**	**Case #2 Pre**	**Case #2 Post**	**Case #3 Pre**	**Case #3 Post**
1.	MQOL	Physical Symptoms (0–10)	6.0	5.5	7.2	8.3	7.0	5.0
		Physical Well-Being (0–10)	7.0	6.0	9.0	9.0	5.0	4.0
		Psychological (0–10)	10.0	10.0	9.4	9.3	10.0	9.5
		Existential (0–10)	9.6	9.7	10.0	10.0	9.3	6.7
		Support (0–10)	10.0	10.0	10.0	10.0	8.5	9.0
		**TOTAL SCORE (0–10)**	**8.5**	**8.2**	**9.1**	**9.3**	**8.0**	**6.8**
2.	LLFDI	Upper Extremity Functioning (0–25)	0	9.0	5.0	0	14.0	20.0
		Basic Lower Extremity Functioning (0–25)	3.0	6.0	6.5	0	5.0	14.0
		Advanced Lower Extremity Functioning (0–25)	9.0	17.0	5.0	5.0	8.0	21.0
		**TOTAL SCORE (0–75)**	**12.0**	**32.0**	**16.5**	**5.0**	**27.0**	**55.0**
3.	ESAS	Pain (0–10)	2.0	2.0	0	1.0	3.0	4.0
		Fatigue (0–10)	4.0	3.0	4.0	3.0	4.0	7.0
		Nausea (0–10)	0.5	0	0	4.0	0	7.0
		Depression (0–10)	0	0	0	0	0	0
		Anxiety (0–10)	0	0	0	2.5	0	0
		Drowsiness (0–10)	0	0	0	0	1.0	6.0
		Appetite (0–10)	0	5.0	0	3.0	1.0	10.0
		Well-Being (0–10)	0	3.0	0	2.0	2.0	4.0
		Dyspnea (0–10)	0	0	0	0	1.0	3.0
4.	BFI	TOTAL Global Fatigue (0–10)	2.0	4.8	0.1	2.3	2.0	6.9
5.	HDS	Authentic Spirit Factor (1–7)	1.0	1.0	1.0	1.0	1.2	2.0
		Comfort Factor (1–7)	3.3	3.3	2.5	3.3	2.0	3.5
6.	Physical parameters	Height (m)	1.6	1.6	1.6	1.6	1.6	1.6
		Weight (kg)	158.8	150.1	84.9	82.5	59.1	60.1
		BMI (kg/m2)	55.8	59.0	34.0	33.0	23.7	24.1
		Resting Blood Pressure (mm Hg)	138/80	112/72	122/76	106/80	148/80	132/90
		Resting Heart Rate (bpm)	86	83	93	88	84	108
7.	8-Foot up-and-go	Number of seconds required	12.4	14.0	5.81	5.44	8.41	10.0
8.	Chair sit-and-reach	Left (number of centimeters)	−30.0	−15.0	+1.0	+1.0	0	−12.0
		Right (number of centimeters)	−33.0	−15.0	+2.0	+2.0	0	−11.0
9.	Arm curl	Left (number of repetitions)	15	12	15	15	18	11
		Right (number of repetitions)	16	12	13	17	14	10
10.	Back scratch	Left (number of centimeters)	−24.0	−31.0	+2.0	+0.5	−27.0	−30.0
		Right (number of centimeters)	−29.0	−32.0	+3.0	+2.0	−24.5	−24.0
11.	Grip strength	Left (kg·feet)	32.0	34.0	24.3	24.5	34.0	25.5
		Right (kg·feet)	31.8	31.8	32.0	31.0	16.0	17.0
12.	30 second chair stand	Number of repetitions	10	8	13	17	15	11
13.	Four test balance scale	Feet together (number of seconds)	10.0	10.0	10.0	10.0	10.0	10.0
		Semi-tandem (number of seconds)	10.0	10.0	10.0	10.0	10.0	10.0
		Tandem (number of seconds)	10.0	0	10.0	10.0	10.0	2.0
		One leg stand (number of seconds)	2.81	0	10.0	10.0	4.0	0
14.	6-minute walk	Total distance (m)	264.0	162.4	467.4	488.7	320.0	250.9

The participant was prescribed a daily walking plan of 5 minutes per day at low to moderate intensity, to progress up to a total of 30 minutes per day at the end of six weeks. All strength exercises were started at 1 set of 8 repetitions, slowly progressing up to 2 sets of 8 repetitions for most exercises. The patient was unable to progress beyond walking 10 minutes per day before experiencing severe fatigue. Modifications were made to the strength exercises, with adoption of seated positions where possible. The participant completed 16 out of the 18 prescribed strength exercise sessions, and experienced no adverse events over the course of the 6-week program.

A summary of the participant’s post-intervention assessment is provided in Table 1. As monitored by the *activ*PAL™ accelerometer, the average number of steps taken over the baseline week was 3714, with an average estimated total energy expenditure of 29.1 MET·hours; post intervention, the average number of steps taken during Week Six was 1471, with an average estimated total energy expenditure of 28.3 MET·hours. The majority of his steps were taken inside his home. It was noted that there was no change in dexamethasone dose over the course of the 6-week program. The patient reported significant total fatigue that likely impacted his endurance and mobility. The patient expressed high satisfaction with the physical activity program and identified one-on-one supervision of the strength training sessions as among its top advantages. The participant indicated that his least enjoyed program aspect was his decline in overall condition despite participating in the physical activity program. In follow-up, the participant passed away 77 days after completing the study.

#### Case 2

A 51 year-old woman was diagnosed with lung cancer and brain metastases. She received a full course of palliative whole brain radiotherapy (WBRT), and daily dexamethasone was initiated. She was recruited from the outpatient radiotherapy unit after completion of WBRT, and a summary of the participant’s baseline assessment is provided in Table 1. Her most common reported physical activity over the past week was walking approximately 30 minutes per day, three times per week.

The participant was prescribed a daily walking plan of 10 minutes per day at low to moderate intensity, to progress up to a total of 40 minutes per day at the end of the six weeks. All strength exercises were started at 1 set of 8 repetitions, slowly progressing up to 2 sets of 10 repetitions for most exercises. After acquiring an upper respiratory tract infection in Week Three, her subsequent dyspnoea and fatigue resulted in the delay in progression of her daily walking program to 20 minutes per day. The participant completed 17 out of the 18 prescribed strength exercise sessions.

A summary of the patient’s post-intervention assessment is provided in Table 1. As monitored by the *activ*PAL™ accelerometer, the average number of steps taken over the baseline week was 11,373, with an average estimated total energy expenditure of 33.3 MET·hours; post intervention, the average number of steps taken during Week Six was 10,868, with an average estimated total energy expenditure of 32.5 MET·hours. The majority of her steps were taken outside the home. It was noted that the participant was being slowly weaned off the dexamethasone over the course of the 6-week program. In follow-up at 60 days post-intervention, the participant had continued her daily walking regimen on her treadmill at home, and was being considered for palliative chemotherapy. Overall, the participant expressed high satisfaction with the physical activity program and identified the home-based location as among its top advantages. The participant indicated her preference for one-on-one training, instead of on her own with the aid of a handbook or DVD.

#### Case 3

A 57 year-old man with hepatitis B was diagnosed with hepatocellular carcinoma post-liver transplant with subsequent liver, lung and bone metastases. He received palliative radiotherapy to the right shoulder and thoracic spine for bony metastatic pain. The patient was recruited to the study post-radiotherapy, and a summary of the patient’s baseline assessment is provided in Table 1. His most common reported physical activity over the past week was walking approximately 60 minutes per day, for three times per week.

The participant was prescribed a daily walking plan of 15 minutes per day at low to moderate intensity, to progress up to a total of 45 minutes per day at the end of the six weeks. All strength exercises were started at 1 set of 8 repetitions, slowly progressing up to 1 set of 12 repetitions for most exercises. After receiving palliative radiotherapy for progressive lymphadenopathy during Week Four, the participant reported worsening nausea and subsequent progression in his exercise prescription was delayed. During Week Four, the participant also exhibited increasing difficulties with balance due to intermittent syncope, and strength exercises were performed in the seated position where possible. The participant completed 14 out of the 18 prescribed strength exercise sessions.

A summary of the participant’s post-intervention assessment is provided in Table 1. As monitored by the *activ*PAL™ accelerometer, the average number of steps taken over the baseline week was 7232, with an average estimated total energy expenditure of 29.1 MET·hours; post intervention, the average number of steps taken during Week Six was 1159, with an average estimated total energy expenditure of 26.9 MET·hours. The majority of his steps were taken inside the home.

Overall, the participant expressed high satisfaction with the physical activity program and identified the strength training component as among its top advantages. In terms of negative experiences, the participant indicated his inability to sustain the aerobic walking component on his own given his increased symptom burden post-radiotherapy. In follow-up, the participant passed away 42 days after completing the study.

## Discussion

The aim of this study was to examine the initial development and pilot testing of a physical activity intervention in patients with advanced cancer receiving palliative care. Based on our pilot survey data, there was a majority preference for home-based, solo interventions, with walking being the most preferred activity [13]. Therefore a modified home-based functional walking program was designed to incorporate the specific physical activity preferences of this sample, and a similar recruitment strategy was adopted.

There are a number of feasibility issues deserving of attention from this study. From our pilot survey study, we were able to recruit 50 patients over a 7 month period [12]; using the same eligibility criteria and local recruitment strategy, however, we were only able to recruit 9 patients over a 6 month period. A total of 504 patients were screened through the RPHCP and CCI outpatient radiotherapy units on behalf of all palliative care research studies that were open for accrual during that 6-month period, however only 15% (96/504) consented to being contacted with regards to this particular study (see Figure 1). In both RPHCP and CCI settings, the first contact was such that the patient’s interest in being contacted by the study coordinator took precedence over obtaining physician-estimated survival; those patients who refused, therefore, may not have fulfilled all eligibility criteria at the time of initial screening.

Of the 96 patients who consented to being contacted by the study coordinator, 53% (51/96) fulfilled all eligibility criteria for this study. Therefore of all patients who consented to being contacted by the study coordinator and who met all eligibility criteria for this study, our accrual rate was 18% (9/51). Locally, this accrual rate is comparable to Hutton et al.’s study of dietary intake in 151 patients with advanced cancer, wherein the authors reported an estimated 21% accrual rate from both the CCI and RPHCP [36]. Elsewhere, Porock et al. reported a recruitment rate of 46% (11/24) in their pilot study of 4-week home-based exercise program in home hospice care patients, with incomplete information as to attrition rates and reasons for withdrawal [11]. Oldervoll et al. reported a recruitment rate of 58% (231/400) in their recent RCT, however the reasons behind refusal to participate were not reported; 36% of the intervention group, versus 23% of the control usual care group, were lost to follow-up primarily due to disease progression [8]. Compared to the 104-day median survival of our pilot survey sample [12], the median survival of the 9 consented participants in this study was 92 days. It is therefore likely that our participants were further along the cancer trajectory than those of Oldervoll et al. [8]. Untimely attrition over a 6-week period in this population with such limited prognosis is not unexpected [37].

From our pilot survey, the majority felt willing and able to participate in a physical activity intervention [13]. The ability to participate in a physical activity program, however, may fluctuate depending on patient-reported symptoms: 69% (35/51) of eligible patients declined consent to the study because of severe symptoms, with fatigue being the most common reported symptom. These findings concur with Mercadante et al.’s prospective study of 400 palliative home care patients with a mean survival of 52 days, wherein there was an increase in fatigue scores over time, with a peak in symptom intensity and frequency at the lowest levels of Karnofsky performance status [38].

Of the 9 patients who enrolled in our study, 6/9 dropped out with the most common reason being admission to acute care for severe symptoms. This rate of attrition is higher when compared to large palliative care trials; Oldervoll et al’s recent RCT reported that 36% of the intervention group, versus 23% of the control usual care group, were lost to follow-up, primarily due to disease progression [8]. In contrast, 5/6 dropouts occurred within 4 weeks of starting our physical activity intervention. Given the shorter median survival of our pilot survey sample, consideration was given to maintain the intervention as long as it was feasible and safe for the patients.

Cases #1 and #2 were obese [BMI ≥ 30 kg/m^2^], with the former meeting the WHO criteria for morbid obesity [BMI ≥ 40.0 kg/m^2^] [39]. The relative proportion of fat to skeletal muscle mass in these patients, however, was not investigated. In a body composition study utilizing computed tomography images of 111 pancreatic cancer patients undergoing palliative treatment, 40% were overweight or obese, and 16% were both sarcopenic and obese; sarcopenic obesity was shown to independently predict survival, and was associated with poorer self-assessed functional status [40]. Although one may postulate that obesity contributed to poor mobility and physical functioning in these cases, further studies are required to elucidate the relationship between sarcopenic obesity, physical functioning and physical activity levels in this population.

In all three cases, increasing symptom burden resulted in the delay in progression in both the aerobic and strength exercise components. There were no reported difficulties with use of the *activ*PAL™ or its generation of data; the number of steps and estimated total energy expenditure, however, decreased significantly over the course of six weeks. Although none of the three participants achieved the target daily walking prescription, all 3 participants were able to continue both aerobic and strength components at reduced levels. Future consideration should be given to a maintenance, rather than progressive, target daily walking prescription given the symptom burden of this patient population.

Currently, there is no recommended minimum level of physical activity for palliative cancer patients [41]; however any amount of physical activity that the patient can tolerate may be better than engaging in no activity at all. Hence one-on-one supervision takes on greater significance in our study, wherein modifications could be made to strength exercises without missing the entire session completely.

On the other hand, one-on-one supervision resulted in the exclusion of potentially eligible participants. Of the 20 eligible patients who were screened from the Department of Symptom Control and Palliative Care and the CCI outpatient radiotherapy units and who consented to being contacted by the study coordinator, 35% (7/20) were unable to participate because they lived out-of-town. While having one-on-one supervision was identified as one of the top advantages by the three presented case reports, the option of a self-directed intervention by means of telehealth approaches, an instructional handbook or video may increase accrual in future pilot trials. Likewise, future consideration should be given to streamlining the number of outcome assessments in view of being less burdensome on this patient population.

Moreover, patients may recognize the difference between a one-time cross-sectional survey on physical activity and a six-week progressive physical activity intervention. Given that the recruitment agencies and processes were identical, one would expect the influence of gatekeeping to be equivalent between this study and our previous pilot survey [12]. Taken together, our results suggest that patients who expressed interest in the idea of physical activity, may have encountered barriers to participating and carrying through with an actual intervention. Eliciting patient barriers to physical activity would therefore be deserving of future research.

Nevertheless, improvements were noted in total MQOL scores in two of the three cases presented. In contrast, two of the three cases showed a decline in physical functioning, as demonstrated by the total LLFDI scores. All three participants shared an overall trend towards worsening ESAS symptom scores, and worsening total BFI global fatigue scores post-intervention. Because of the small sample size, it is not possible to distinguish whether these effects were secondary to the physical activity program or to progression in the underlying cancer; as shown in Headley et al’s pilot RCT of a seated exercise program in stage IV breast cancer patients [42], a slowing of the inevitable decline in fatigue and quality of life scores may be a realistic interventional goal which would account for the changes seen in our case series.

Although this small sample precludes drawing conclusions on intervention effects or determining sensitivity of outcome measures, our case series provides rationale for future feasibility studies. With respect to the local recruitment strategy, further characterization of the screened patient population, including exploration of the reasons for declining consent to be contacted for research, would aid in defining which subgroup would most benefit from an intervention. Recruitment and retention may be improved by opening enrolment to advanced cancer patients irrespective of clinician-estimated prognosis. Further modifications, such as shortening the duration of the intervention, examining the effects of aerobic or strength components separately, and including an option for self-directed programming, may also optimize recruitment and retention.

## Conclusions

This case series demonstrates the challenges of actual participation in a physical activity intervention in end stage cancer patients. Although our pilot survey sample reported a strong interest in physical activity, a similar recruitment strategy for a pilot intervention yielded higher than expected attrition and drop-outs due to symptom severity and disease burden. Further feasibility research is required on the role of physical activity in patients with advanced cancer receiving palliative care.

## Competing interests

None of the authors have any potential conflicts of interest.

## Authors’ contributions

SSL and KSC conceived and designed the study, and drafted the manuscript. SL conducted the study, data collection and data analysis. SW and VB participated in study conception and design, and helped to draft the manuscript. All authors read and approved the final manuscript.

## Authors’ information

SSL is supported by a full-time Roche Fellowship in Translational Cancer Research from the Alberta Cancer Foundation. KSC is supported by the Canada Research Chairs Program.

## Pre-publication history

The pre-publication history for this paper can be accessed here:

http://www.biomedcentral.com/1472-684X/12/22/prepub

## Supplementary Material

Additional file 1: Appendix 1 Modified home-based functional walking (FW) program. Click here for file

Additional file 2: Appendix 2 Guidelines for the modified home-based functional walking (FW) program. Click here for file

Additional file 3: Appendix 3 Objective Physical Function Measures (listed in the order of testing). Click here for file
