# Supplementary material for: Home-based functional walking program for advanced cancer patients receiving palliative care: a case series
Source: BMC Palliat Care. 2013 May 11;12:22. doi: 10.1186/1472-684X-12-22 (PMC3655875; doi:10.1186/1472-684X-12-22)
Supplement: Additional file 1: Appendix 1 — Modified home-based functional walking (FW) program. [file 1472-684X-12-22-S1.pdf]

**Appendix 1: Modified home-based functional walking (FW) program**

| | Exercise | Purpose | Target muscle / joint | Examples of equipment | Position |
|---|---|---|---|---|---|
| 1. | Chin to chest | Range of motion | Cervical spine | None | Seated |
| 2. | Chin to shoulder | Range of motion | Cervical spine | None | Seated |
| 3. | Shoulder shrugs | Range of motion | Shoulder joint | None | Seated |
| 4. | Arm swing | Range of motion | Shoulder joint | None | Seated |
| 5. | Rowing | Range of motion | Shoulder and elbow joints | None | Seated |
| 6. | Forward hugs | Range of motion | Lower back and upper arms | None | Seated |
| 7. | Up and down leg march | Range of motion | Hip joint | None | Seated or standing |
| 8. | Out and in leg march | Range of motion | Hip joint | None | Seated or standing |
| 9. | Foot forward and backward | Range of motion | Knee joint | None | Seated or standing |
| 10. | Toe point/flex and Wrist flex/extend | Range of motion | Ankle and wrist joints | None | Seated |
| 11. | Ball squeeze | Resistance training Grip strength | Wrist and forearm muscles | Thera-band® hand exercisers | Seated |
| 12. | Chest press | Resistance training | Pectoralis major, triceps and deltoids | Thera-bands® | Seated |
| 13. | Rowing | Resistance training | Latissimus dorsi, trapezius, biceps and deltoids | Thera-bands® | Seated |
| 14. | Biceps curl | Resistance training | Biceps | Neoprene dumbbells | Seated |
| 15. | Triceps curl | Resistance training | Triceps | Thera-bands® or neoprene dumbbells | Seated |
| 16. | Sit to stand | Resistance training | Quadriceps, hamstrings and gluteals | None | Seated and standing |
| 17. | Knee flexion | Resistance training | Hamstrings | Ankle cuff weights | Seated or standing |
| 18. | Toe raises | Balance retraining | Tibialis anterior | None | Standing |
| 19. | Heel raises | Balance retraining | Gastrocnemius and soleus | None | Standing |
| 20. | Swan | Stretching | Pectoralis major, deltoids and biceps | None | Seated |
| 21. | Zipper stretch | Stretching Flexibility | Latissimus dorsi and triceps | Thera-bands® | Seated or standing |
| 22. | Tibia touch | Stretching Flexibility | Hamstrings and spinal erectors | None | Seated |
| 23. | Outer thigh stretch | Stretching | Hip abductors | None | Seated |
| 24. | Quadriceps stretch | Stretching | Quadriceps and tibialis anterior | None | Seated or standing |
| 25. | Calf stretch | Stretching | Gastrocnemius and soleus | None | Seated or standing |
