# Supplementary material for: Home-based functional walking program for advanced cancer patients receiving palliative care: a case series
Source: BMC Palliat Care. 2013 May 11;12:22. doi: 10.1186/1472-684X-12-22 (PMC3655875; doi:10.1186/1472-684X-12-22)
Supplement: Additional file 2: Appendix 2 — Guidelines for the modified home-based functional walking (FW) program. [file 1472-684X-12-22-S2.pdf]

Appendix 2: Guidelines for the modified home-based functional walking (FW) program

Program Components	General Guidelines
1. Muscle strengthening	Whole body resistance training with special emphasis on lower body muscle strengthening for transferring, standing, walking and prevention of falls<br><br>Balanced resistance training of opposing muscle groups:<br>- Chest/back muscles<br>- Biceps/triceps<br>- Quadriceps/hamstrings<br>- Tibialis anterior/gastrocnemius and soleus<br><br>Maintenance of proper technique, breathing and positioning on range of motion prior to use of resistance equipment
2. Balance retraining	Whole body balance training with special emphasis on ankle dorsiflexors and plantar flexors for recovery of balance<br><br>Maintenance of posture and balance using body weight alone prior to adding resistance equipment
3. Intensity	Goal of low to moderate intensity measured using Borg's ratings of perceived exertion (RPE) (0-10)
4. Resistance	Progression by:<br>• Supported to unsupported positions<br>• Range of motion or body weight to addition of resistance equipment<br>• Start with the lightest resistance equipment and upwards<br>• Increasing number of sets/repetitions prior to progression of resistance level<br><br>Reduce workload if:<br>• Increased pain, fatigue or shortness of breath post exercise<br>• Muscle pain or soreness > 48 hours<br><br>Terminate session if:<br>• Excessive pain, fatigue or shortness of breath post exercise
5. Sets	Progression by:<br>• Start with one set of 8 repetitions per exercise<br>• RPE ≤ 3 consistent for ≥ 2 sessions prior to increasing number of repetitions by 2 until maximum of 15 repetitions per set<br>• Once one set of 15 repetitions achieved, advanced to two sets of 8 repetitions
6. Frequency	3 days per week, on non-consecutive days
