# Supplementary material for: Home-based functional walking program for advanced cancer patients receiving palliative care: a case series
Source: BMC Palliat Care. 2013 May 11;12:22. doi: 10.1186/1472-684X-12-22 (PMC3655875; doi:10.1186/1472-684X-12-22)
Supplement: Additional file 3: Appendix 3 — Objective Physical Function Measures (listed in the order of testing). [file 1472-684X-12-22-S3.pdf]

Appendix 3: Objective Physical Function Measures (listed in the order of testing)

Physical Function Test	Purpose	General Description
1. 8-Foot up-and-go	Agility and dynamic balance	Number of seconds required to get up from a seated position, walk 8 feet, turn and return to a seated position
2. Chair sit-and-reach	Lower body flexibility	Number of centimeters between extended fingers and tip of toe, when leg extended and hands reaching towards toes from a seated position at the front of a chair
3. Arm curl	Upper body strength	Number of bicep curls completed in 30 seconds holding a hand weight (5 lbs for women, 8 lbs for men)
4. Back scratch	Upper body flexibility	Number of centimeters between extended middle fingers, when one hand is reaching over the shoulder and the opposite hand up the middle of the back
5. Grip strength	Hand and forearm strength	Number of kilogram-feet measured on handheld dynamometer (average of 2 measurements per side)
6. 30 second chair stand	Lower body strength	Number of full stands completed in 30 seconds from a seated position
7. Four test balance scale	Static balance	Number of seconds up to 10 seconds each that participant is able to sustain 1) feet together stand, 2)semi-tandem stand, 3) tandem stand and 4) one leg stand
8. 6-minute walk	Aerobic endurance	Number of meters that participant can walk in 6 minutes around a 45.7 meter course
